# Pre-Hospital Orthopaedic Trauma Care in a Developing Country During COVID-19 Pandemic

**DOI:** 10.5704/MOJ.2111.027

**Published:** 2021-11

**Authors:** AS Soni, SK Garg, A Singhal

**Affiliations:** Department of Orthopaedics, Government Medical College and Hospital, Chandigarh, India

Dear editors,

Pre-hospital care has been shown to reduce the mortality in trauma patients even without sophisticated measures. Despite of this, the system is not established well by developing countries^1^. The COVID-19 pandemic has further worsened the situation in an unprecedented way. While other scientific councils have developed new guidelines during current pandemic, no literature is available regarding pre-hospital orthopaedic trauma care in developing countries^2^. We studied the status of pre-hospital orthopaedic trauma care during COVID-19 pandemic at a tertiary health centre in the northern part of India. This centre caters for patients from within the city as well as from outside. Like most of the developing countries there is no established referral system to refer patients from peripheral areas to a higher health care centre.

We studied all the patients received in emergency department between 25th March 2020 and 31st January 2021 having orthopaedic traumatic injuries. A total of 1044 patients were evaluated for pre-hospital orthopaedic trauma care. Seven hundred and forty-eighth of 1044 patients (71.6%) came from outside the city, and 347 (46.4%) of all patients who were received from outside city came directly without any referral. Of 401 patients received from outside city with referral, only 276 (68.8%) were with splinted fractures, 309 (77.0%) had received analgesics and 285 (71.0%) had received intravenous fluids from local doctors. This highlights the trend of trauma patients being shifted directly to tertiary health centre without getting primary treatment from local physician. This direct shifting delays the most basic treatment like intravenous fluid, immobilisation of fractured part, dressing of wounds and antibiotics. Even referred patients are not getting adequate pre-hospital care.

Since the COVID-19 situation seems far from over, the pre-hospital trauma care will continue to be a challenge. Many hospitals are changed to dedicated COVID-19 treatment centres leaving no option of local treatment for other patients. Decontamination of transport vehicle and inability to maintain social distance at the site of accident complicates the situation^3^. Furthermore, at the site of accident, fear of getting COVID-19 prevent people from helping the injured. However, certain things like co-ordination between primary, secondary and tertiary health care level can be improved. Community level participation has been proven to be an effective measure to improve trauma care^4^. This holds true during COVID-19 pandemic also. Educating and encouraging people for community level contributions without breaking COVID-19 protocols is the need of the hour.
